# Detecting shifts in nonlinear dynamics using Empirical Dynamic Modeling with Nested-Library Analysis

**DOI:** 10.1371/journal.pcbi.1011759

**Published:** 2024-01-05

**Authors:** Yong-Jin Huang, Chun-Wei Chang, Chih-hao Hsieh

**Affiliations:** 1 National Center for Theoretical Sciences, Taipei, Taiwan; 2 Institute of Oceanography, National Taiwan University, Taipei, Taiwan; 3 Institute of Fisheries Science, National Taiwan University, Taipei, Taiwan; 4 Research Center for Environmental Changes, Academia Sinica, Taipei, Taiwan; 5 Institute of Ecology and Evolutionary Biology, National Taiwan University, Taipei, Taiwan; Universidade Federal do Parana, BRAZIL

## Abstract

Abrupt changes in system states and dynamical behaviors are often observed in natural systems; such phenomena, named regime shifts, are explained as transitions between alternative steady states (more generally, attractors). Various methods have been proposed to detect regime shifts from time series data, but a generic detection method with theoretical linkage to underlying dynamics is lacking. Here, we provide a novel method named Nested-Library Analysis (NLA) to retrospectively detect regime shifts using empirical dynamic modeling (EDM) rooted in theory of attractor reconstruction. Specifically, NLA determines the time of regime shift as the cutting point at which sequential reduction of the library set (i.e., the time series data used to reconstruct the attractor for forecasting) optimizes the forecast skill of EDM. We illustrate this method on a chaotic model of which changing parameters present a critical transition. Our analysis shows that NLA detects the change point in the model system and outperforms existing approaches based on statistical characteristics. In addition, NLA empirically detected a real-world regime shift event revealing an abrupt change of Pacific Decadal Oscillation index around the mid-1970s. Importantly, our method can be easily generalized to various systems because NLA is equation-free and requires only a single time series.

## Author summary

Abrupt shifts in system dynamics, referred to as regime shifts, are common in natural systems and pose significant challenges for system management and risk assessment. Accurately detecting the change point that separates pre- and post-regime shift periods is crucial, as the data collected after regime shift can be more informative to forecast future system states. While numerous methods have been proposed to tackle this issue, identifying change points in chaotic systems remains difficult, in which regime shift signals can be concealed by chaotic dynamics. To address this issue, we propose Nested-Library Analysis (NLA), a machine learning method rooted in a data-driven, nonparametric framework for nonlinear dynamical systems, known as Empirical Dynamic Modeling (EDM). NLA effectively identifies the change points from time series data, even when the shifts in dynamics are nearly imperceptible due to chaotic behavior, surpassing the existing change point detection methods. As such, our method offers a generic solution for revealing the timing of regime shifts in a various type of dynamical system.

## 1. Introduction

In the Earth’s history, abrupt shifts from one regime to another have often been recorded in various ecosystems around the world [1], and such transition is difficult to reverse [2]. For instance, kelp forests in Australia were replaced by seaweed turfs within just a few years, with no sign of recovery despite of abating climate stressors [3]; nitrate concentrations in the River Thames acutely increased and stayed intractably high in spite of sustained management intervention [4]; biological communities in the Central Baltic Sea ecosystem have not recovered from an abrupt shift occurred around 1990 [5]. Such abrupt shifts, termed as regime shifts, can proceed very fast and bring devastating socio-economic impacts.

In essence, regime shifts bear the same dynamical mechanisms, from the viewpoint of bifurcation theory [6–9]. This viewpoint suggests that complex systems (e.g., ecosystems) have alternative attractors [10,11]—the sets of system states possessing their own resilience (i.e., ecosystems regimes). A transition can occur among these regimes, due to increased perturbation (such as environmental stresses) that changes bifurcation parameters or creates shocks to system variables [2,8,12–16]. Although this viewpoint has been suggested for decades [17] and widely accepted as the origin of regime shifts with plenty of empirical and theoretical supports [6–8,10–13,18–20], it has rarely been considered in existing methods used to detect the real-world regime shifts.

A wide variety of approaches have been proposed to detect regime shifts in time series [21]; however, quantitative evaluation of the change point in dynamical system (i.e., the timing of regime shift) remains highly uncertain in existing methods for three reasons. First, the existing methods used to detect change points have unclear linkage with the theoretical viewpoint regarding a regime shift as a transition between attractors in dynamical systems. A good proportion of these methods are to determine change points according to shifts in statistical characteristics, such as mean, skewness, information entropy, to name but a few (See [21] and references therein). However, shifts in statistical properties are not necessarily caused by a transition between attractors, *vice versa*. Second, not all the state variables exhibit clear shifting patterns in time series observations during the regime shift [22]. That is, apparent shifts only present in some variables in the complex systems, whereas clear bifurcation patterns might be hidden in some system variables. In practical sense, it is possible that apparent shifts only present in the variables that are not observable or out of consideration [23]. Last, many of the methods are developed for the systems exhibiting equilibrium dynamics, but a challenging aspect is that natural systems are often not in balance, i.e., non-equilibrium systems, of which attractors are not necessarily fixed points [24–26].

**Fig 1:**
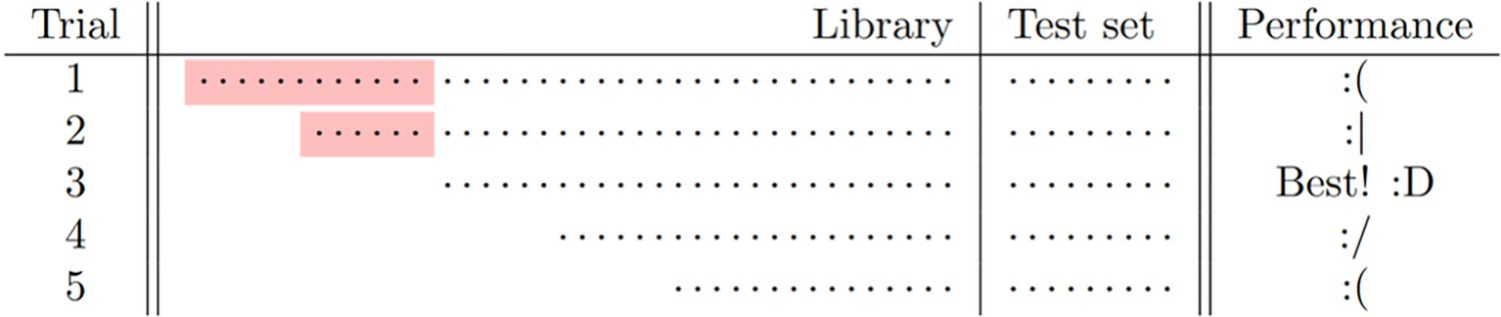
The main idea of Nested-Library Analysis. We fix an end of a time series as the test set to evaluate the out-of-sample forecast skill, and repeatedly chop off the data from the other end. Here, each dot represents the data point of a time series observed at each moment. Observational data following a regime different from that of the test set is colored in red, i.e., misleading data points to predict the test set.

To provide a generic regime shift detection method, we proposed a novel method called Nested-Library Analysis (NLA). This method is developed based on a main argument that observational data of one regime would interfere with the reconstruction of another regime. Thus, our analytic framework is to monitor the quality of attractor reconstruction while sequentially eliminating the data points collected from different regimes (as **Fig 1**). To realize this idea in practice, we evaluate the quality of attractor reconstruction from the forecast skill in Empirical Dynamic Modeling (EDM) [27,28], which is a data-driven modeling method for empirical attractor reconstruction based on Takens’ embedding theorem [29]. Indeed, EDM-based methods have been applied in previous studies of regime shift [30]. For instance, a previous algorithm [31] was proposed to test whether a transition is significant between two pre-chosen time series segments. However, applying this statistical test requires prior information about existing change point that divides a time series into two segments, which differs from the proposed NLA algorithm that aims to quantitatively determine the change point of regime shift and circumvents the aforementioned problems of previous change point models (CPMs), a class of algorithms that detect location and/or scale shifts based on shifts in statistical properties. Apparently, detecting regime shifts from the viewpoint of attractor reconstruction strongly connects to the theoretical view regarding regimes as attractors in dynamical systems. Moreover, attractor reconstruction by means of Takens’ embedding requires neither the full information about the governing equation nor the complete data of the whole system, but only needs the time series data of a single variable. Especially, EDM-based methods enable to analyze chaotic systems in general [32]. Overall, our analysis presents a novel analytical framework to empirically detect the timing of regime shift (change point) based on attractor reconstruction. We first mathematically formalize the problem and specify the assumptions, then formulating the process of NLA. Next, we test the efficacy of NLA in analyzing a synthetic dataset from a chaotic food chain model coupled with nutrient cycling that underwent a regime shift, and then compare the performance of NLA with CPMs. Finally, we empirically apply NLA in revealing the change point of a real-world regime shift in Pacific Decadal Oscillation (PDO) index occurred around the mid-1970s.

## 2. Methods

### 2.1. Background

We assume that there is a discrete-time system *σ*_*ω*_: *X*×ℕ_0_→*X* defined by the governing equation *X*_*t*+1_ = *f*(*X*_*t*_)∈*X*, where the family ℕ_0_ of non-negative integers represents the timeline and *ω*∈*Ω* stands for external factors (e.g., environmental conditions). This setting does not lose the generality, because a time series is always a discretized sequence of observation, and the time unit of sampling can be scaled to exactly 1. Here, we say *ω* is a vector of external factors as its evolution is independent of the system state *X*, i.e., *ω*_*t*+1_ = *g*(*ω*_*t*_) and $\frac{\partial g}{\partial X}\equiv 0$.

For the sake of argument, we suppose that the system experiences a regime shift in which governing equation *f*_*ω*_ (i.e., the dynamics) abruptly changes. Therefore, our attempt is to detect a change point *τ* that satisfies

Xt+1=fωt(Xt)=f(Xt,ωt)withfωt≈{F0F1t<τotherwise, (1)

where $\frac{\partial f}{\partial \omega }$ is non-trivial and two functions *F*_*i*_: *X*→*X* (*i* = 0,1) are distinct. This discontinuous approximation of dynamical system aims to highlight the presence of regime shift and the timing of its occurrence (i.e., change point *τ*). Moreover, the discontinuous approximation requires the assumptions that variations in *f*_*ω*_ within regime are ignorable compared to the substantial changes caused by regime shift despite of the continuous changes in *f*_*ω*_ and *ω*_*t*_ even within each regime. For simplicity, we assume that there exists either no or exactly one change point in the time series data because multiple regime shift events rarely occur within a short period of observation. Moreover, NLA algorithm is applied in a moving-window manner in which each time window is so short that hardly includes more than one change point. In brief, our goal is to detect an abrupt change point *τ* in *f*_*ω*_ given a chaotic time series $\{x_{t}{\}}_{t\in {\mathbb{N}}_{0}}$ sampled from $\{X_{t}{\}}_{t\in {\mathbb{N}}_{0}}$ (namely, *x*_*t*_ = *ϕ*(*X*_*t*_) for some smooth observation function *ϕ*: *X*_*t*_∈*X*↦*x*_*t*_∈ℝ).

### 2.2. The framework of Nested-Library Analysis

Our first task is to reconstruct the attractors for the underlying governing equations from the time series data. A strategy that can be easily brought out is to use delay-coordinate embedding approaches based on Takens’ theorem. Despite that the governing equation of the system varies along time, the time series data are still embeddable in the light of a higher hierarchy as *σ*^♯^: (*X*×*Ω*)×*N*_0_→(*X*×*Ω*) of which governing equation is

(Xt+1ωt+1)=(f(Xt,ωt)g(ωt))≕f˜(Xt,ωt) (2)

and $\{x_{t}{\}}_{t\in {\mathbb{N}}_{0}}$ is sampled from $\{{(X}_{t},\omega_{t}){)}_{t\in {\mathbb{N}}_{0}}$. In words, the time series can still be embedded when considering the observations sampled from a larger dynamical system, which consists of the interested system as well as external factors. However, this would require a larger embedding dimension and thus be relatively unfavorable for kernel methods due to data noises [33]. The question in focus is that change in the governing equation (or parameters of the governing equation) can lead to a regime shift; then, how to detect the change point? Our motivation is to use performance of forecast skill as a criterion for detecting the change point *τ*. Specifically, the attractor reconstructed from time series data within a single regime should exhibit better forecast skill, in comparison to that from time series data across two regimes (**Fig 1**).

To detect the change point, we propose an algorithm named Nested-Library Analysis (NLA). The main idea of NLA is to monitor the performance (i.e., forecast skill) of the trained model while trimming the library dataset (i.e., the time series data used to reconstruct the attractor for forecasting) to reduce those data points from different regimes/attractors. A common way of evaluating out-of-sample forecasting performance is to divide a dataset into a library (i.e., a training set) and a test set. However, the data points included in the library set are not equally informative, and some data points can even interfere with model training. In the case of regime shift, putting together the data points collected from different attractors interfere with the reconstruction of distinct attractors. Therefore, if these data that interfere with model training can be continuously removed from the library set, then the performance of the trained model should be improved; in contrast, if data with correct information diminishes, the prediction skill will decrease. In brief, the spirit of NLA is to monitor the performance of the trained model while the library is shrinking. With the above argument, we implement this idea to a prediction method assumed to have the following properties:

**[A1]** Richer data with *correct* information renders better prediction performance, and

**[A2]** The more *misleading* information the training set contains, the worse the forecast skill is obtained.

Here, a collection of data points (i.e., the library set) is said to offer *correct*/*misleading* information if it helps/disturbs to train a model for the dynamics governing the test set. With properties **[A1]** and **[A2]**, our framework of NLA aims to detect the dynamical change point *τ* in time at which *misleading* information is completely removed but preserve the maximal amount of *correct* information. We note that [A1] is an arguably property of many machine learning methods [34], and thus the framework of NLA is more sensitive to incorporating prediction methods that satisfies [A2].

Our algorithm can start the detection of change point *τ* from either of the end or the beginning of the time series (cf. pseudocode given in S1 Text). To present the key idea, we may first suppose that we have a time series ${\left\{x_{t}\right\}}_{t=0}^{R}$ and launch the NLA algorithm from the left-hand side (the algorithm can be symmetrically employed as demonstrated in Sec. 3.2). For any *l*∈[0, *L*], we can use ${\left\{x_{t}\right\}}_{t=l}^{L}$ as the library set to train a model and obtain the prediction ${\left\{y_{t}^{\left(l\right)}\right\}}_{t=L+1}^{R}$ for the test set ${\left\{x_{t}\right\}}_{t=L+1}^{R}$. One can notice that the library set shrinks from the left as *l* goes larger, and we can see how the forecast skill varies with *l* by considering the root-mean-square error (RMSE)

$${Ɛ}_{0}(l)≔\sqrt{\sum_{t=L+1}^{R}\frac{({x_{t}-y_{t}^{\left(l\right)})}^{2}}{R-L}}.$$

Then, according to the two properties **[A1]** and **[A2]**, we can determine whether misleading or correct information is being eliminated by tracking the descendance or ascendance of the prediction error, respectively. Hence, with *τ* being the change point, we have

dƐ˜0dl(t)<0⇐by[A2]t<τdƐ˜0dl(t)>0⇐by[A1]t>τ(Discriminant)

where ${\tilde{\varepsilon }}_{0}$ is a smoothed curve of ε_0_ created using a Gaussian filter (which involves convolution with a Gaussian probability density function). This preserves a moving-average-like meaning and ensures that ${\tilde{Ɛ}}_{0}\in C^{\infty }(R)$. Consequently, we obtain the estimation ${\hat{\tau }}_{0}$ for *τ* according to the discriminant. To alleviate the computation efforts, one can let *l* vary in an arithmetic sequence (e.g., *l* = 0,3,6,9,…) of a larger common difference *D*_*skipstep*_. Analogously, we can derive an estimation ${\hat{\tau }}_{n}$ by assuming the input time series to be ${\left\{x_{t}\right\}}_{t=n}^{L}$ instead of ${\left\{x_{t}\right\}}_{t=0}^{L}$; then, in an ensemble fashion, we take the median of ${\hat{\tau }}_{n}$’s as the final output $\hat{\tau }$.

According to the aforementioned argument, the library in NLA algorithm is required to shrink, forming a nested sequence of library sets. The use of a nested sequence of library sets enables NLA to quantitatively reveal the change point, which differs from general cross-prediction framework applied across multiple time series without delicate arrangement of library sets [35]. When any two libraries were not nested (i.e., one library is not a subset of the other), the algorithm will not provide reliable results. For instance, suppose that we have two time series segments, $L_{\alpha },L_{\beta }\subseteq {\left\{x_{t}\right\}}_{t\in N_{0}}$ such that $\left(L_{\alpha }\backslash L_{\beta })\neq \emptyset \neq (L_{\beta }\backslash L_{\alpha }\right)$, it is difficult to tell whether their differences in prediction skill is a consequence of including more correct/misleading information or simply because they capture distinct parts of the attractor.

To launch NLA, we choose S-map based on Empirical Dynamic Modeling (EDM) as the prediction method [27,28], which is a delay-coordinate embedding approach satisfying **[A1]** and **[A2]**. The idea of S-map is to train local weighted auto-regressive model around the predictee on the reconstructed attractor, where the weight of each library data point is negatively related to its distance from the predictee with an exponential decay manner [27,28]. Such a method can usually be more powerful than simply averaging the evolution of *k*-nearest neighbors [36]. In NLA, S-map is only used for one-step forward forecast (i.e., predicting the future value at *t*+1) throughout the test set, which avoids long-term forecast that has been found more challenging for chaotic dynamics [27]. Like most manifold-based learning methods, S-map clearly possesses the property **[A1]** since an attractor is reconstructed better as the data points get richer and denser, especially around the parts in which only a few data points originally located (e.g., rare extreme events). Moreover, S-map also satisfies **[A2]** because each data point in the library set renders a weighted contribution to model forecast even if the data point actually lies in another attractor (i.e., misleading information). More detailed S-map procedure is provided in S2 Text.

## 3. Results

### 3.1. A food chain in a suddenly changed environment

To test NLA, we first examine a simulated model where a known shift in an external factor (regime variable) triggers abrupt changes in the governing equation of a chaotic system (**Table A** in S3 Text). For simplicity, we let the governing equation of this chaotic system linearly depend on the regime variable that manifests an abrupt shift in mean value. Specifically, a three-species food chain given by [37] is selected as our target system,

$$\{\begin{array}{lll} \dot{x}=x(1-x) & -f_{1}(x)y & \\ \dot{y}=-d_{1}y & +f_{1}(x)y & -f_{2}(y)z\\ \dot{z}=-d_{2}z & & +f_{2}(y)z \end{array}$$ (3)

with type II functional responses $f_{i}(u)=\frac{p_{i}u}{1+q_{i}u}$ in the predator-prey interactions between species *y* and *x* (*i* = 1) and between species *z* and *y* (*i* = 2), where *p*_*i*_ and *q*_*i*_ determine attack rate and handling time, respectively. Here, *d*_1_ and *d*_2_ are natural mortality of consumer *y* and *z*, respectively. To include a regime variable, we modify this three-species food chain to include lake nutrient cycle [38]

$$\dot{N}=a-bN+\frac{cN^{m}}{N^{m}+1}≕g(N)$$ (4)

The three terms stand for the input from river loading (*a*), loss process (-*bN*), and recycling from sediments or consumers, respectively, where *c* and *m* determine the scale and reversibility of eutrophication, respectively. In addition, we let the mortality rate *d*_1_ and the interaction coefficient between *x* and *y*, i.e., *p*_1_ in type II functional response *f*_1_(*u*), linearly depend on the value of *N* (**Table A** in S3 Text). When the bifurcation parameter, loading *a*, increases smoothly, it triggers a regime shift in *N*. Such a shift was caused by local bifurcation with hysteresis property [39,40], which is consistent with the generic framework of critical transition proposed previously [8]. As such, *N* is defined as a regime indicator of this model. To render the synthesis data more verisimilitude, process errors (i.e., the stochastic perturbance) and measurement errors are involved. Due to stochasticity, a shift in *N* does not always occur at a certain moment. Hence, in each numerical experiment, we crop a 1000-step time series data such that the maximal change rate of *N* reaches at *t* = 300, defined as *τ* (i.e., the change point). Details of numerical simulations can be found in S3 Text.

**Fig 2:**
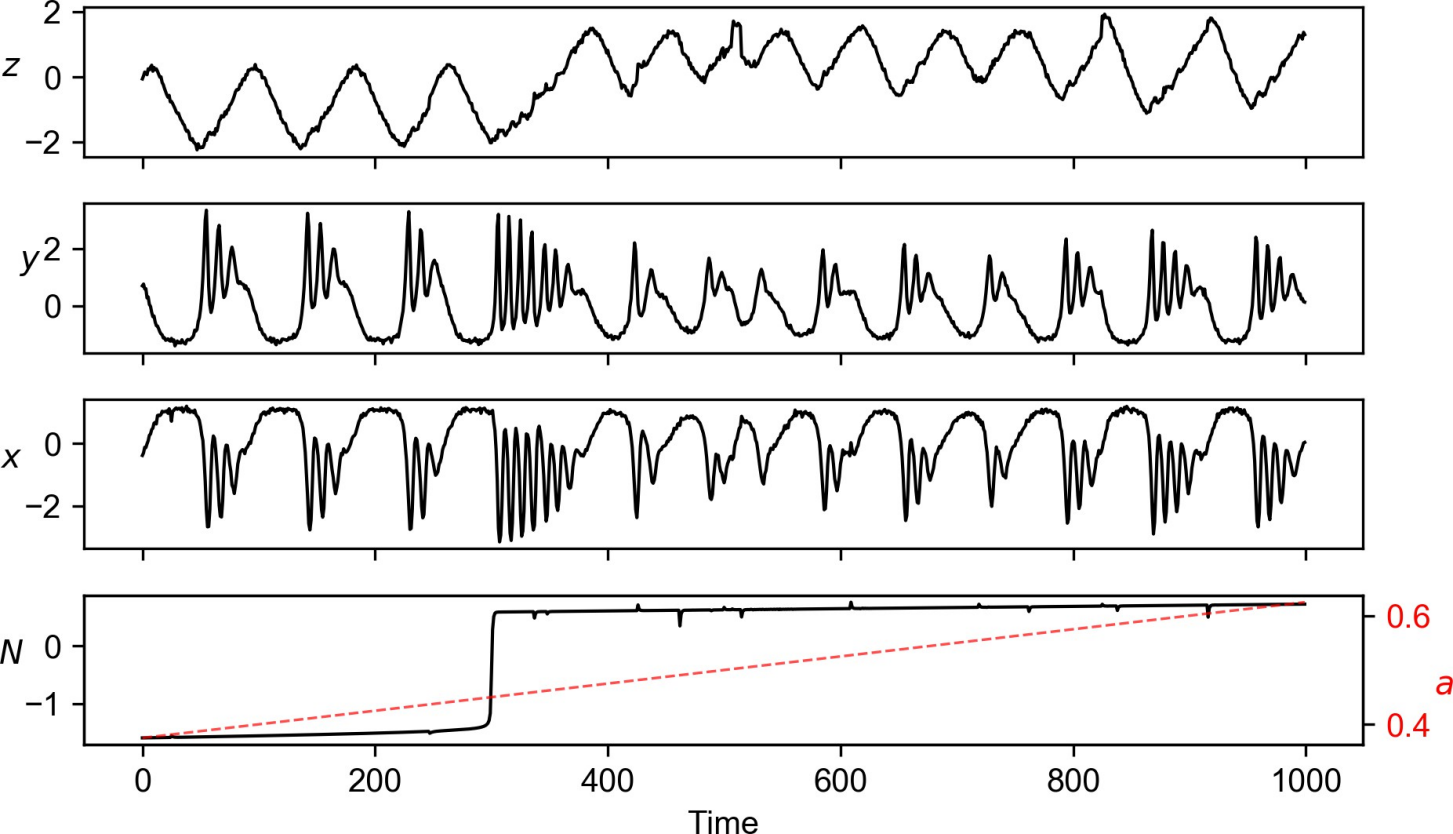
An example of simulated model time series with settings described in S3 Text. The shift pattern is clearly exhibited by *N* whereas the timing is not quite distinguishable in *z*. It is even harder to tell that the variables *y* and *x* experience a regime shift from their time series, despite that they are exactly the variables of which equations directly depend on the regime indicator *N*. The process errors and measurement errors are set with *ρ* = 0.05 and *σ*^2^ = 0.05.

**Fig 3:**
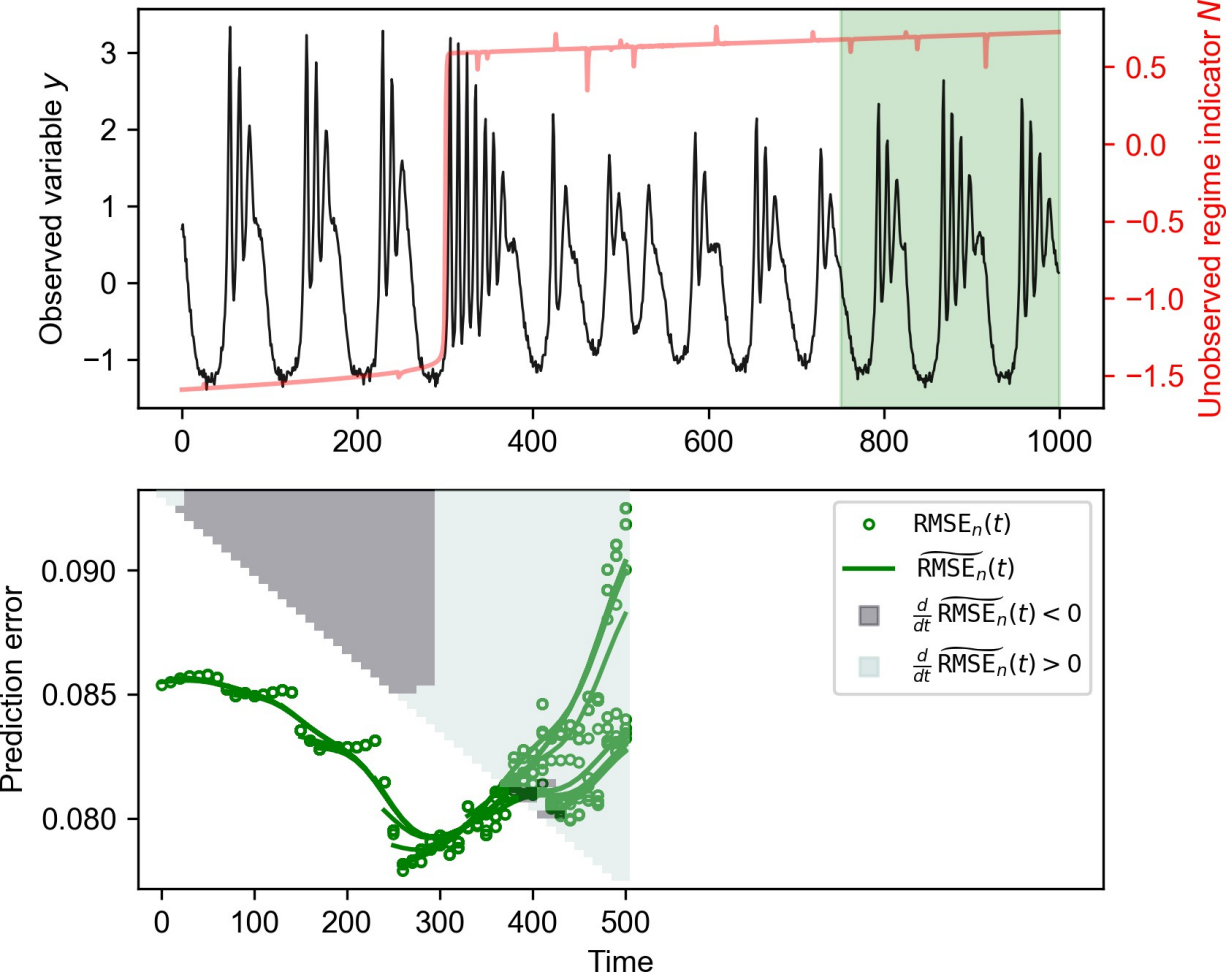
NLA applied to the model time series obtained in the simulation shown in Fig 2. The input time series ${\left\{y_{t}\right\}}_{t=0}^{999}$ together with the unobserved regime indicator *N* (red line) is shown in the upper panel. The last quarter ${\left\{y_{t}\right\}}_{t=750}^{999}$ is used as the test set, and the rest part of the time series is used as the shrinking library sets. The scatter plot in the lower panel illustrates the forecast RMSEs with the smoothened green curves ${\tilde{E}}_{l}$ obtained by applying Gaussian filters (with the Gaussian function *σ*^2^ = 3) to $E_{l}’s$ for *l* = 0, 10, 20, …., respectively. NLA is terminated at *l* = 500 at which the valley-shapedness of RMSE curve has been clearly displayed. To better visualize the sign of the derivative of ${\tilde{E}}_{l}’s$ at each time point *t*, a grid triangle is plotted in the lower panel. Each row of grids represents the sign of the derivative of ${\tilde{E}}_{l}’s$ along time, with dark gray and light blue indicating for negative and positive derivatives, respectively. Namely, the *t*-th grid (counted from the left) of the *l*-th row (counted downwardly) denotes whether $\frac{d{\tilde{E}}_{l}}{dt}(t)$ is positive or negative. Results show that NLA succeeds to detect the change and estimate the change point as $\hat{\tau }$ = 300.

An example of our pedagogical model is shown in **Fig 2**. Clear shift patterns are not visible in the time series data of *x* and *y*, even though *x* and *y* receives direct influences from *N* (**Table A** in S3 Text). In contrast, a shift in mean is presented in *z*, even though *z* is not directly affected by *N*; however, the time of the shift in mean is ambiguous to tell. To illustrate the efficacy of NLA method, we may assume that we have nothing else but the time series data of the variable *y*. Using the last quarter of the time series as the test set and the root mean square error as the prediction error, letting the library shrink from the left side as *l* = 0, 10, 20, 30,…, we obtain the result shown in **Fig 3**. The convex shapes of the curves indicate that there is an abrupt shift in the dynamics of *y*, and the change point is given as $\hat{\tau }=300$ nicely. In addition, NLA has reasonably low false negative and false positive rate in detecting the change point (0 and 0.08, respectively in S4 Text).

**Fig 4:**
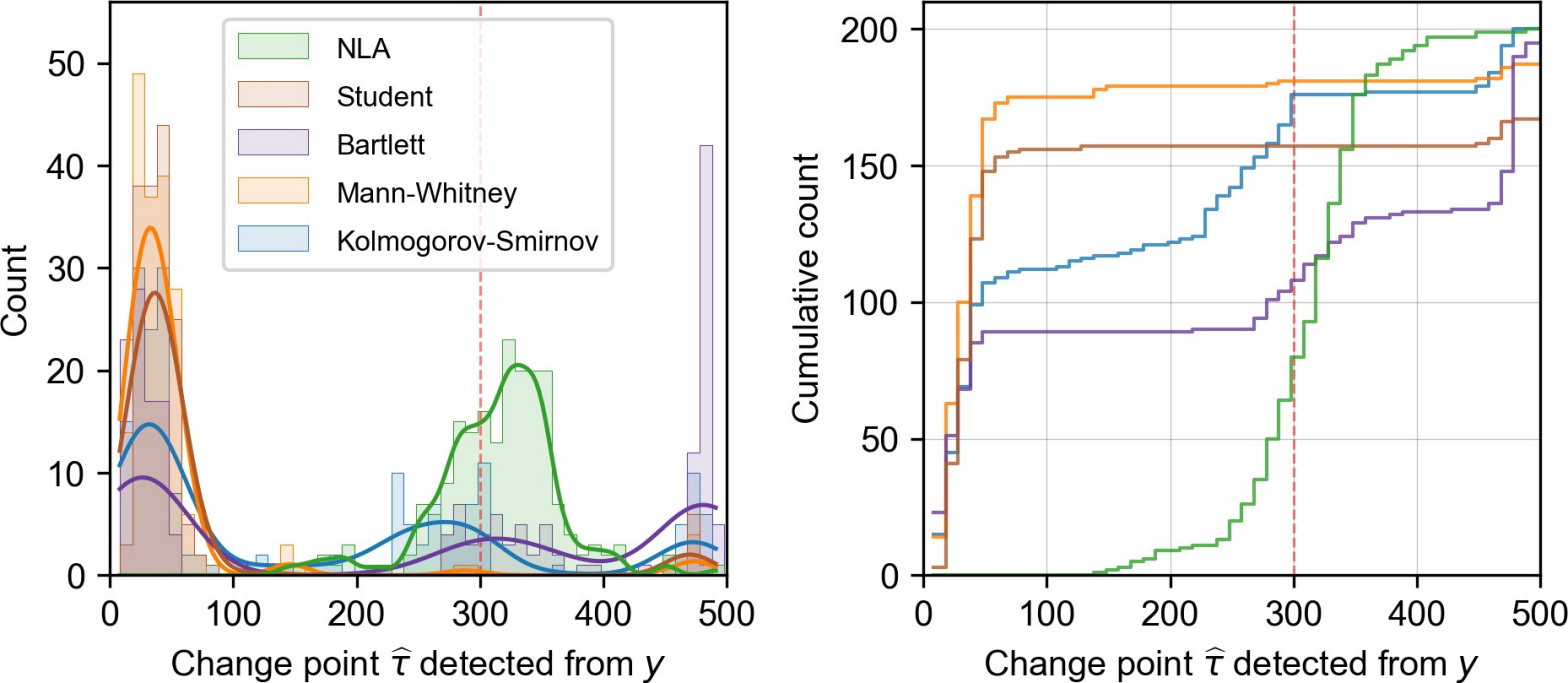
The comparison between NLA and other methods. The left panel illustrates the distributions of change points determined by NLA and CPM based on Student-*t* test, Bartlett test, Mann–Whitney test, and Kolmogorov–Smirnov test, when analyzing the *y* time series using 200 replicates generated from the food-chain model. The right panel presents empirical cumulative distributions of the estimated change point obtained by NLA and CPM methods.

To further validate whether NLA outperforms traditional approaches designed to identify shifts in statistical properties, we compare our NLA with a suite of methods known as the Change Point Model (CPM). We use CPM as a representative of existing regime shift detection methods because the framework of CPM incorporates a wide variety of test statistics [41,42]. All the CPM analyses were conducted using the R package **cpm** (version: 2.2). Here, we present two parametric CPMs based on Student-*t* test and Bartlett test for detecting shifts in mean and variance, respectively. In addition, two nonparametric CPMs, Mann–Whitney test and Kolmogorov–Smirnov test, are also applied to detect changes in the mean and probability distribution of the time series data. Then we applied both NLA and CPM analysis in 200 time series replicates obtained from the simulations stated in S3 Text. Our analysis (**Fig 4)** clearly indicates that the change points detected by NLA are closer to the desired *τ* = 300 than those derived from any of the CPMs. Analyzing the distribution of detected change points reveals that CPM easily pulls the false alarm, especially in the beginning of the time series (*t*<100), or even fail to detect any change point in many cases. NLA also outperformed the CPMs on analyzing the variable *x* and *z* (see details in S5 Text), suggesting that the performance of NLA is robust to the choice of system variable (**Table 1**).

**Table 1:** Summary of results from different methods. The true change point is fixed as *τ* = 300 for each experiment. Each method is applied to each of variables for 200 times.

	On X				On Y				On Z			
	count	median	mean	std	count	median	mean	std	count	median	mean	std
NLA	193	300.0	276.347	92.697	200	320.0	312.100	50.979	197	280.0	280.381	71.119
Student-*t*	161	44.0	118.478	114.243	165	39.0	61.000	98.871	200	328.5	324.770	29.767
Bartlett	182	46.5	195.363	213.673	190	276.0	227.489	198.999	200	367.5	274.385	206.202
Mann-Whitney	195	46.0	128.697	133.036	185	36.0	54.503	83.345	200	328.5	325.135	28.957
Kolmogorov-Smirnov	198	61.5	170.606	158.139	200	47.5	145.535	144.649	200	175.0	167.700	82.456

### 3.2. Detection of regime shift in the Pacific Decadal Oscillation

**Fig 5:**
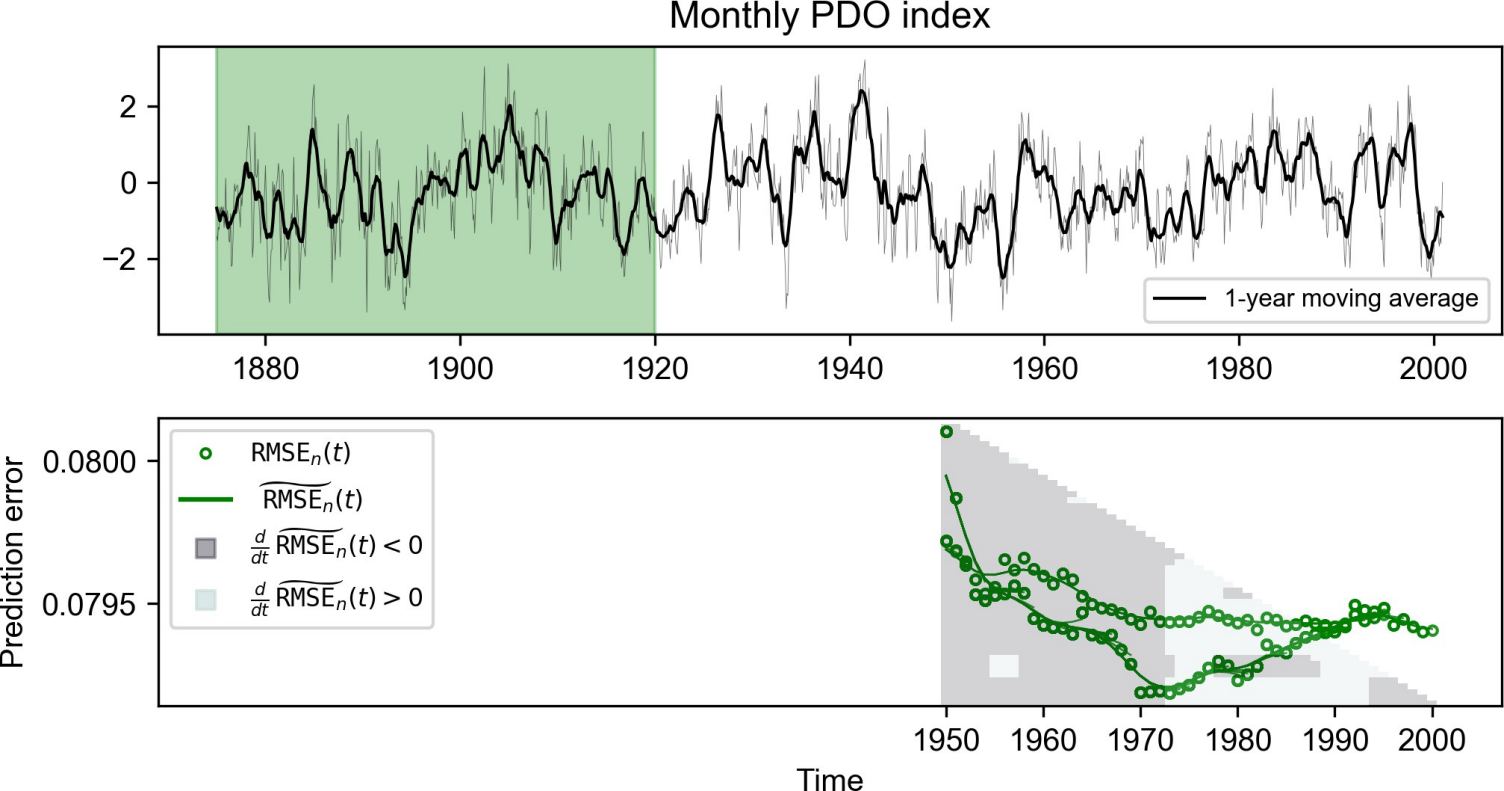
A demonstration of NLA with monthly PDO index data shows that a shift occurs in its dynamics around $\hat{\tau }$ = 1973. Data points of the time series before 1920 are set as the test set, and the rest of the data points are used as the library set. When the library set shrinks from the right, the RMSE becomes lower until historical data points after the mid-1970s are excluded; in comparison, the model performance gets worse when the algorithm starts to remove data points before the mid-1970s.

To demonstrate our approach for empirical data, NLA is applied to the Pacific Decadal Oscillation (PDO) index, which is a key indicator of climate variability. Many studies have indicated the presence of a regime shift in PDO in mean or the positive/negative phases around the 1970s; however, we would like to investigate whether there is a structural change in the underlying dynamics of the climate system. The monthly reanalysis PDO data are publicly available from National Oceanic and Atmospheric Administration (NOAA) [43], and we take the one-year moving average of the time series from 1875 to 2000 prior to the analysis (**Fig 5**). Note that the presence of secular trends in empirical time series, in any, likely biases the result of tracking prediction error, and thus needs to be removed prior to NLA (i.e., detrending time series [28]). However, due to lack of clear trend, detrending was not performed for the analysis of PDO and model data.

For the purpose of detecting the change in its dynamics during the second half of the 20th century, time series data before 1920 are reserved as the test set, and the rest of the data are used as the library set from the right. When the data points are sequentially removed from the library from the right-hand side, the RMSE decreases until the right boundary of the library set reaches the mid-1970s (**Fig 5**). According to our argument, the forecast skill improved in spite of the decreasing amount of historical data points, indicating that the removed data points were generated by another governing equation. Based on the result of NLA, we conclude a shift in the dynamics of the PDO index around $\hat{\tau }$ = 1973. This finding is supported by the empirical evidence of the change in the climatology of Alaska [44], South America [45], central equatorial Pacific [46], and the properties of El Niño [47]. Compared to CPMs, only Mann-Whiteny method obtained the change point estimate around mid-1970s (change point estimates, Student-*t*: 1957; Bartlett: 1950; Mann-Whitney: 1976; Kolmogorov-Smirnov: 1952).

## 4. Discussion

Natural systems in the changing environment may undergo abrupt changes in dynamics. It is demanding to identify regime shifts that are often concealed due to chaos, data noises, or the unobservable variables, etc. Here, we have provided a data-driven method called Nested-Library Analysis based on EDM. Our numerical experiment on a chaotic food chain coupled with nutrient cycling shows that NLA accurately detects the occurrence of regime shifts from any one of the system variables. Such robustness shall be attributed to the generality of Takens’ embedding theorem, which guarantees attractor reconstruction from any of system variables. For comparison, CPMs as conventional detection methods targeting the changes in statistical properties give decent estimates only for the variable *z*, the only system variable not directly affected by the regime variable *N* but exhibiting a clear shift in its empirical distribution. In contrast, CPMs provide poor estimates of change point for variables *x* and *y*, as *x* and *y* do not exhibit noticeable changes in statistical properties (**Fig 2**). Our example shows that abrupt changes in the governing equation might not be necessarily accompanied with apparent shift patterns in the empirical distribution, i.e., shifts in mean and variance cannot serve as a generic signal of regime shifts. In other words, the existing CPMs confirm the significance of shifting patterns that have been observed; while NLA, by leveraging its theoretical linkage with attractor identification, enables to unveil those hidden shifts in dynamical systems and serve as a generic regime shift detection method regardless of the presence of shifting pattern in time series. Indeed, NLA can be applied to a generic framework of regime shift [8] that considers not only abrupt shift but also flickering in variable states (S6 Text).

It is noteworthy that the analytical framework NLA can potentially be extended to detect multiple change points by implementing moving-windows computation used in general CPM methods. Specially, the curve of prediction error shown in **Fig 3** can move along the time axis by applying NLA to the moving library and test sets. As such, multiple change points can be revealed by the appearance of multiple prediction optima at different times. Nevertheless, implementing moving-window computation requires more delicate parameter selection (e.g., [30]), and thus requires more detailed investigations when applying NLA to analyze long time series underwent multiple regime shifts, e.g., multiple glacier periods revealed in paleoclimatic records.

**Fig 6:**
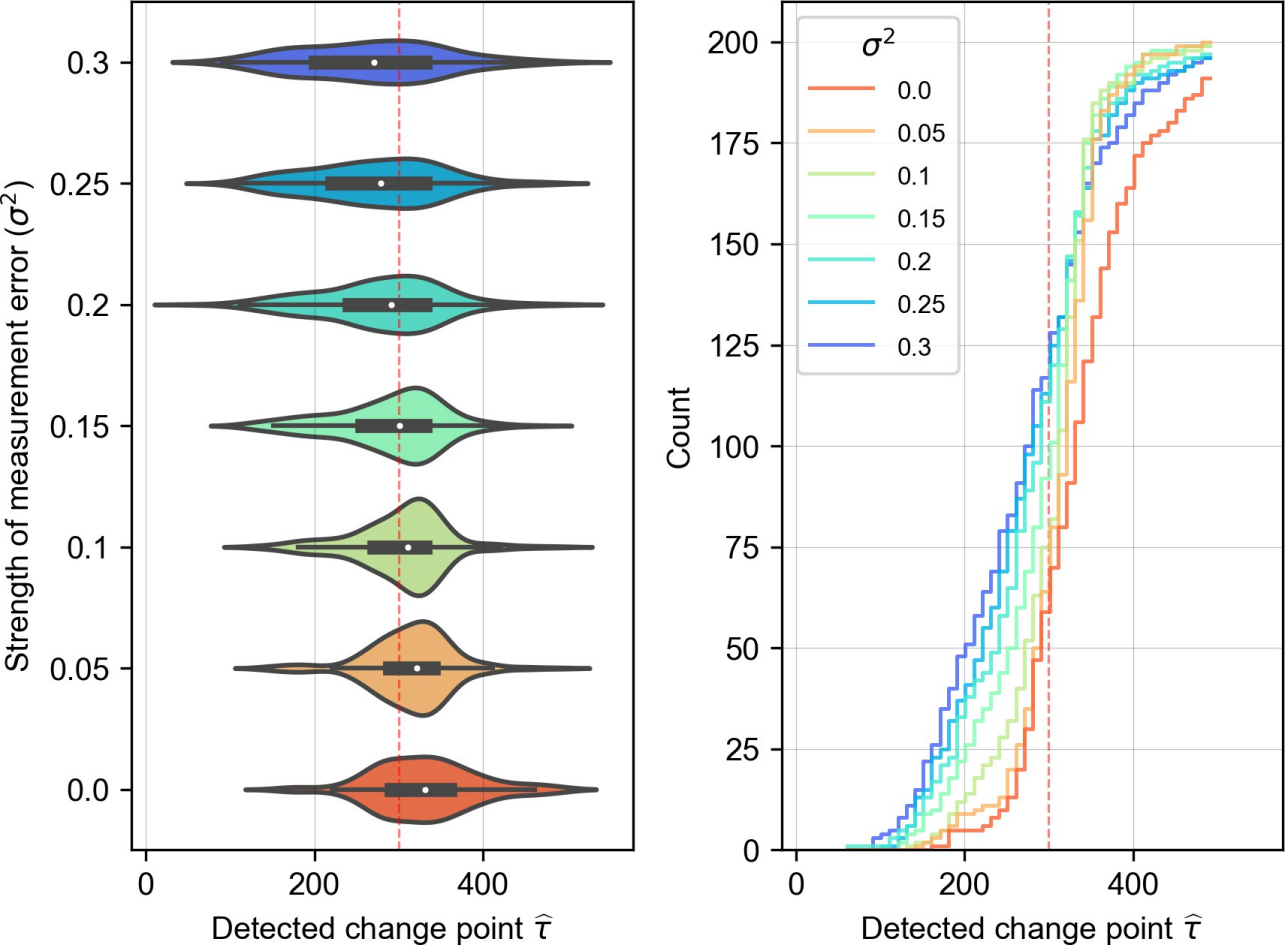
To investigate the sensitivity of our detection method to data noises, we repeat the experiment as described in the Section 3.2 but with measurement errors in different levels. The violin plot on the left panel suggests that NLA performs better when the data are slightly noisy. In particular, the accuracy and the precision are optimized when the measurement error scale *σ*^2^ is 0.15 and 0.1 respectively. The right panel presents empirical cumulative distributions of the estimated change point obtained under different noise levels.

For practical purposes, we also investigate the efficacy of NLA in the presence of data noise. We find that the performance of NLA is optimized in the presence of minor data noises (**Fig 6**). In details, we repeat the numerical experiment of the food chain model and incorporate the simulated dataset with different levels of measurement errors, i.e., white Gaussian noises $\varepsilon_{t}∼N(0,\sigma^{2})$, based on the detailed procedure provided in S3 Text. It is not surprising that the performance deteriorates as the signal-to-noise ratio declines. Interestingly, however, the performance of NLA is not optimal when applying it to a noise-free dataset. The suboptimal in noise-free condition can be explained upon better understandings of the S-map method implemented in NLA. Specifically, detecting the change point by NLA requires the inclusion and then exclusion of the impacts of misleading data collected from the regime differing from test set (i.e., false neighbors in state space). However, if the contribution of misleading data can be down-weighted very effectively by S-map, which usually happens in noise-free condition, the prediction errors caused by including misleading data in library set can be greatly reduced and leads to a less accurate change point estimation in NLA. Nonetheless, the case of too-weak data noise is less of a problem in practice as real-world data are generally noisy and minor white noises can be additionally introduced as shown in the previous method [48]. However, strong noise reduces the efficacy of NLA in empirical cases because noise blurs the identification of neighborhood structures (namely, the topology) of reconstructed attractors. Consequently, strong noise leads to unclear valley-shapedness of $\tilde{E}$, resulting in a less accurate estimation of change point. A possible solution for analyzing noisy data is to apply multiview embedding (i.e., an ensemble learning of S-map) that is known to be more effective in analyzing noisy data, but this approach requires multivariate time series data. In addition, considerable error margin of change point estimates shown in **Fig 6** is likely caused by the uncertainty in determining the valley of prediction error. In most cases, the valley can be well-identified from the sign changes in the first derivative of smoothened error curve. However, such criterion also results in a few extremities in noisy systems, especially when the error curve is less sharp and contains certain noise.

A problem one may come across in practice is the choice of the test set. The test set should be long enough to capture the topological structure of an attractor, so that the forecast skill can be evaluated over the entire attractor. Nonetheless, a longer test set means the less room for the library set in conducting the sequential elimination of nested library in NLA. There is yet to be an answer for what is the best trade-off. Whereas, a rule of thumb is to have the test set not less than two cycle times (or, more generally, the Poincaré recurrence statistics [49]). One should keep in mind that the existence of a test set is required to evaluate the performance of out-of-sample forecasts, which is associated with the choice of the test set. To circumvent the problem of choosing a test set, one may consider alternative approaches that does not necessitate a test set. For example, methods based on unsupervised learning can infer the quality of attractor reconstruction by judging the level of attractor collapsing, e.g., the waving product method and the filter-factor algorithm. Nevertheless, the NLA algorithm implemented by EDM can be applied to the time series data without long record or dense sampling interval. Indeed, our results based on half of the sample size in model time series (S7 Text) remains qualitatively similar as the findings based on full sample size, indicating the advantage of EDM-based methods on analyzing sparser time series [50]. Moreover, the result of NLA is also robust to the selection of test set based on either the first or last quarter (S8 Text), provided that the change point is included in the library set. If the potential change point is difficult to be recognized in chaotic systems, we suggest applying the NLA twice, taking each of ends of time series data as the test set.

Insights into historical events are vital to studying the underlying mechanisms of a natural system. Being able to detect the timing of regime shifts is a pressing concern. To overcome the problems and limitations of existing approaches that rely on analyzing statistical properties of time series data, we have proposed a new data-driven method Nested-Library Analysis (NLA). NLA performs better than the existing methods at determining the timing of abrupt changes of dynamical systems even in the problematic case, in which the changes in time series are hidden by noises or complex chaotic dynamics. We anticipate the development of this novel detection method can unveil the critical events that threaten system sustainability and thus facilitate the management and restoration of natural systems.

## Supporting information

S1 Text Pseudocodes of Nested-Library Analysis. (DOCX)

S2 Text Sequential locally weighted global linear map (S-map). (DOCX)

S3 Text The setting for the simulations of the food chain model. (DOCX)

S4 Text NLA analysis for model time series with and without the true change point. (DOCX)

S5 Text Application of NLA on different system variables of the food chain model. (DOCX)

S6 Text Efficacy of NLA in analyzing time series underwent flickering regime shift. (DOCX)

S7 Text Efficacy of NLA in sparse time series. (DOCX)

S8 Text Robustness of NLA to the selection of test set located at either ends of the time series. (DOCX)
