# Supplementary material for: Detecting shifts in nonlinear dynamics using Empirical Dynamic Modeling with Nested-Library Analysis
Source: PLoS Comput Biol. 2024 Jan 5;20(1):e1011759. doi: 10.1371/journal.pcbi.1011759 (PMC10795988; doi:10.1371/journal.pcbi.1011759)
Supplement: S1 Text — (DOCX) [file pcbi.1011759.s001.docx]

**Supplementary Materials for**

Detecting shifts in nonlinear dynamics using Empirical Dynamic Modeling with Nested-Library Analysis

Yong-Jin Huang, Chun-Wei Chang*, and Chih-hao Hsieh

*Correspondence to: [cwchang@ntu.edu.tw](mailto:cwchang@ntu.edu.tw)

**This supplement file includes:**

**S1 Text**

# S1 Text Pseudocodes of Nested-Library Analysis

| **Algorithm 1:** Nested-Library Analysis, checking the left part of the time series |
| --- |
| **Data:** Time series ${\{x_{t}\}}_{t=0}^{L}$ as the library set, ${\{x_{t}\}}_{t=L+1}^{R}$ as the test set, and a number $D_{skipstep}\mathbb{\in N}$  **Result:** An estimated time $\hat{\tau}\in[0,L)$ of a regime shift if it exists |
| **for** $\left( n=0;n<L;n=n+D_{skipstep} \right)$ **do**   \| /* Step 1. Monitor the prediction error curve ${\tilde{\mathcal{E}}}_{n}$ */  $\theta_{n}\leftarrow\underset{\theta}{arg min} \left\{ \sum_{t=L+1}^{R} {(y_{t}-x_{t})}^{2} \vert y_{t}: \text{the prediction of }x_{t}\text{ given by S-map }\left( {\{x_{t}\}}_{t=n}^{L}; \theta\right) \right\}$;  // the optimal parameter for S-map to predict ${\{x_{t}\}}_{t=L+1}^{R}$ with ${\{x_{t}\}}_{t=n}^{L}$.  **for** $\left( l=n;l<L;l=l+D_{skipstep} \right)$ **do**   \| ${\{y_{t}^{(l)}\}}_{t=L+1}^{R}\leftarrow\text{the prediction of }{\{x_{t}\}}_{t=L+1}^{R}\text{ given by S-map }\left( {\{x_{t}\}}_{t=l}^{L}; \theta_{n} \right)$;  $ℇ_{n}(l)\leftarrow\sqrt{\sum_{t=L+1}^{R} \frac{{(x_{t}-y_{t}^{(l)})}^{2}}{R-L}}$; \| \| --- \|   **end**  /* Step 2. Find the change point $\tau_{n}$ */  $\tilde{ℇ}_{n}\leftarrow\text{ the smoothened }ℇ_{n}\text{ by Gaussian filter;}$  **if** $\tilde{ℇ}_{n}$ is valley-shaped **then**   \| $\hat{\tau}_{n}\leftarrow\underset{t}{arg min} \tilde{ℇ}_{n}\left( t \right);$ \| \| --- \|   **else**   \| $\hat{\tau}_{n}\leftarrow\text{NaN;}$ \| \| --- \|   **end** \| \| --- \| --- \| --- \| --- \|   **end**  /* Step 3. Vote for the output */  $\hat{\tau}\leftarrow\text{median of }{\{\hat{\tau}_{n}\}}_{n}$;  **return** $\hat{\tau}$; |

| **Algorithm 2:** Nested-Library Analysis, checking the right part of the time series |
| --- |
| **Data:** Time series ${\{x_{t}\}}_{t=L+1}^{R}$ as the library set, ${\{x_{t}\}}_{t=0}^{L}$ as the test set, and a number $D_{skipstep}\mathbb{\in N}$  **Result:** An estimated time $\hat{\tau}\in(L+1,R]$ of a regime shift if it exists |
| **for** $\left( n=R;n>L+1;n=n-D_{skipstep} \right)$ **do**   \| /* Step 1. Monitor the prediction error curve ${\tilde{\mathcal{E}}}_{n}$ */  $\theta_{n}\leftarrow\underset{\theta}{arg min} \left\{ \sum_{t=0}^{L} {(y_{t}-x_{t})}^{2} \vert y_{t}: \text{the prediction of }x_{t}\text{ given by S-map }\left( {\{x_{t}\}}_{t=L+1}^{n}; \theta\right) \right\}$;  // the optimal parameter for S-map to reconstruct ${\{x_{t}\}}_{t=0}^{L}$ with ${\{x_{t}\}}_{t=L+1}^{n}$.  **for** $\left( l=n;l>L+1;l=l-D_{skipstep} \right)$ **do**   \| ${\{y_{t}^{(l)}\}}_{t=0}^{L}\leftarrow\text{the prediction of }{\{x_{t}\}}_{t=0}^{L}\text{ given by S-map }\left( {\{x_{t}\}}_{t=L+1}^{l}; \theta_{n} \right)$;  $ℇ_{n}(l)\leftarrow\sqrt{\sum_{t=0}^{L} \frac{{(x_{t}-y_{t}^{(l)})}^{2}}{L}}$; \| \| --- \|   **end**  /* Step 2. Find the change point $\tau_{n}$ */  $\tilde{ℇ}_{n}\leftarrow\text{ the smoothened }ℇ_{n}\text{ by Gaussian filter;}$  **if** $\tilde{ℇ}_{n}$ is valley-shaped **then**   \| $\hat{\tau}_{n}\leftarrow\underset{t}{arg min} \tilde{ℇ}_{n}\left( t \right);$ \| \| --- \|   **else**   \| $\hat{\tau}_{n}\leftarrow\text{NaN;}$ \| \| --- \|   **end** \| \| --- \| --- \| --- \| --- \|   **end**  /* Step 3. Vote for the output */  $\hat{\tau}\leftarrow\text{median of }{\{\hat{\tau}_{n}\}}_{n}$;  **return** $\hat{\tau}$; |

**Remark.** The valley-shapedness of curves is determined by the discriminant in Sec. 2.2.
