# Supplementary material for: Detecting shifts in nonlinear dynamics using Empirical Dynamic Modeling with Nested-Library Analysis
Source: PLoS Comput Biol. 2024 Jan 5;20(1):e1011759. doi: 10.1371/journal.pcbi.1011759 (PMC10795988; doi:10.1371/journal.pcbi.1011759)
Supplement: S2 Text — (DOCX) [file pcbi.1011759.s002.docx]

**Supplementary Materials for**

Detecting shifts in nonlinear dynamics using Empirical Dynamic Modeling with Nested-Library Analysis

Yong-Jin Huang, Chun-Wei Chang*, and Chih-hao Hsieh

*Correspondence to: [cwchang@ntu.edu.tw](mailto:cwchang@ntu.edu.tw)

**This supplement file includes:**

**S2 Text**

**S2 Text Sequential locally weighted global linear map (S-map)**

For a chosen dimension $m$, a delay term $\tau$ (which does not stand for the dynamical change point here), and a given time series ${\{x_{t}\}}_{t}$, the attractor is reconstructed as ${\{X_{t}\}}_{t}$ with

$$X_{t} :=\left( 1, x_{t}, x_{t-\tau}, \ldots, x_{t-\left( m-1 \right)\tau} \right)\in\mathbb{R}^{m+1}\text{ for each moment }t\text{,}$$

Where the trivial first entry is set for constant terms. Specifically, the forecast for a predictee $Y_{t} =\left( 1, y_{t}, y_{t-\tau}, \ldots, y_{t-\left( m-1 \right)\tau} \right)=:(Y_{t}\left( 0 \right), Y_{t}\left( 1 \right), \ldots, Y_{t}(m))$ is going to be given in a locally linear fashion as

$\hat{y_{t+1}}= \sum_{j=0}^{m} C_{t}(j)Y_{t}(j)$,

where $C_{t}(j)$’s are trained by using the historical points from the library set ${\{X_{t}\}}_{t}$. For each predictee $Y_{t}$ (with a fixed $t$), the way of training linear combination coefficients $C_{t}$ is to fit

$$B=AC$$

with $B_{s}=w(\left\| X_{s}-Y_{t} \right\|)\cdot x_{s+1}$ and $A_{sj}=w(\left\| X_{s}-Y_{t} \right\|)\cdot X_{s}(j)$, where the weighting kernel is given by

$w\left( d \right)=e^{-(\theta d/\bar{d})}$.

Here, $\bar{d}$ is the mean distance from $Y_{t}$ to its $k$ ($=m+1$ as default) nearest neighbors.

Parameters that have to be determined at first, in practice, are chosen to be the one optimizing the out-of-sample forecast. As mentioned in Sec. 2.2, the parameters $m$ and $\theta$ are determined by the largest library when NLA is applied.
