# Supplementary material for: Detecting shifts in nonlinear dynamics using Empirical Dynamic Modeling with Nested-Library Analysis
Source: PLoS Comput Biol. 2024 Jan 5;20(1):e1011759. doi: 10.1371/journal.pcbi.1011759 (PMC10795988; doi:10.1371/journal.pcbi.1011759)
Supplement: S3 Text — (DOCX) [file pcbi.1011759.s003.docx]

**Supplementary Materials for**

Detecting shifts in nonlinear dynamics using Empirical Dynamic Modeling with Nested-Library Analysis

Yong-Jin Huang, Chun-Wei Chang*, and Chih-hao Hsieh

*Correspondence to: [cwchang@ntu.edu.tw](mailto:cwchang@ntu.edu.tw)

**This supplement file includes:**

**S3 Text**

**S3 Text The setting for the simulations of the food chain model**

The occurrence of process errors, of which arrival times are denoted by $T_{k}$'s, is a Poisson process (with parameter $\lambda=0.01$). At an arrival time $T_{k}$, for every variable we perturb it by scaling it with a random variable that follows a uniform distribution *U*(1−*ρ*, 1+*ρ*) for some $\rho$>0. Long-tailed distributions such as Gaussian distribution are not chosen in order to prevent the system from experiencing multiple regime shifts within a short time. White Gaussian noises, *ε_t_* ~ *N*(0, *σ*^2^)
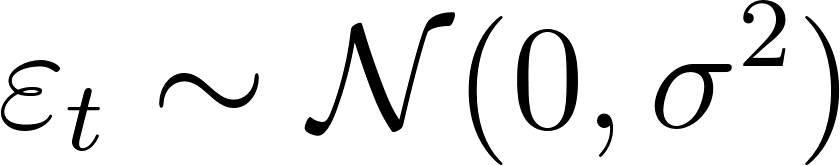
, are added as measurement errors onto the model dataset after the simulation based on **Table A** and data normalization, except for the regime indicator *N* which is not involved in NLA. Note that the magnitude of *σ*^2^ can be considered as the noise level that varied in the sensitivity analysis presented in **Fig 6**.

A community is simulated by the explicit Runge-Kutta method of order 5(4) for 5000 time steps, sampled once every 7 time steps (the sample rate is set arbitrarily). The change point $\tau$ is empirically determined as the moment when the absolute value of change of rate of $N$ reaches its maximum, and we choose the 1000-step time series such that $\tau=300$ for testing NLA. To obtain the sampling distribution of change point estimates (**Fig 4**), we simulated 200 replicates of model time series for NLA and CPM analysis. Here, 1 /*σ*^2^ can be considered as the signal-to-noise ratio.

| \| Network \| Equations and the parameters \| \| \| --- \| --- \| --- \| \| 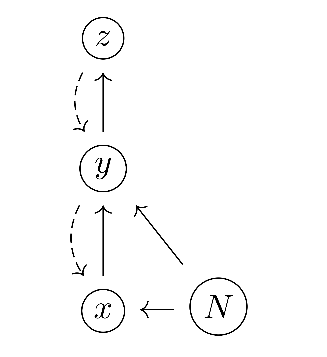 \| $\dot{N}=a-bN+\frac{cN^{m}}{N^{m}+1}$ \| $(b=0.8,m=8,c=1)$ \| \| $\dot{x}=x\left( x-1 \right)-f_{1}y$ \|  \| \| $\dot{y}=-d_{1}y+f_{1}y-f_{2}z$ \|  \| \| $\dot{z}=-d_{2}y+f_{2}z$ \| $(d_{2}=0.01)$ \| \| $d_{1}=c_{0}N+c_{1}$ \| $(c_{0}=0.75,c_{1}=3.7)$ \| \| $f_{1}=(c_{2}N+c_{3})\cdot\frac{x}{1+q_{1}x}$ \| $(c_{2}=0.05,c_{3}=0.4, q_{1}=3.25)$ \| \| $f_{2}=p_{2}\cdot\frac{y}{1+q_{2}y}$ \| $(p_{2}=0.1,q_{2}=2)$ \| \| $a=c_{4}t+c_{5}$ \| $(c_{4}=0.375,c_{5}=0.25,0\leq t\leq5000)$ \| |
| --- | --- | --- | --- | --- | --- | --- | --- | --- | --- | --- | --- | --- | --- | --- | --- | --- | --- | --- | --- | --- |
| **Table A:** The settings of the simulations for the pedagogical example in the Section 3.1. |
