# Supplementary material for: Detecting shifts in nonlinear dynamics using Empirical Dynamic Modeling with Nested-Library Analysis
Source: PLoS Comput Biol. 2024 Jan 5;20(1):e1011759. doi: 10.1371/journal.pcbi.1011759 (PMC10795988; doi:10.1371/journal.pcbi.1011759)
Supplement: S4 Text — (DOCX) [file pcbi.1011759.s004.docx]

**Supplementary Materials for**

Detecting shifts in nonlinear dynamics using Empirical Dynamic Modeling with Nested-Library Analysis

Yong-Jin Huang, Chun-Wei Chang*, and Chih-hao Hsieh

*Correspondence to: [cwchang@ntu.edu.tw](mailto:cwchang@ntu.edu.tw)

**This supplement file includes:**

**S4 Text**

**S4 Text** **NLA analysis for model time series with and without the change point**

To present the correctness of NLA analysis in detecting the change point, we generated 400 time series replicates of variable *y* stated in Appendix C that present no clear shifting pattern even under regime shift, among which 200 replicates have no change point and the other 200 have a change point at *t*=300. Both noise strength and library/testing sets are the same with the main results shown in Figure 3. The results were summarized in a confusion matrix presented in **Table A**, where false positive rate and false negative rate are 0.08 (16 out of 200 replicates) and 0 (0 out of 200 replicates), respectively. The problem of minor false positive rate might be further improved by finely controlling the smoothness of RMSE curve using more sophisticated criteria (e.g., generalized cross validation GCV) to avoid misidentification of RMSE valley from false sign changes in the first derivative of RMSE curve.

|  |  | **Predicted condition** | |
| --- | --- | --- | --- |
|  | Total  200 + 200 = 400 | Change point detected | No change point detected |
| **Actual condition** | One change point 200 | True Positive 200 | *False Negative* 0 |
|  | No change point  200 | *False Positive* 16 | True Negative 184 |

**Table A:** The confusion matrix of NLA algorithms based on analyzing 400 time series replicates, considering scenarios with and without true change point.
