# Supplementary material for: Detecting shifts in nonlinear dynamics using Empirical Dynamic Modeling with Nested-Library Analysis
Source: PLoS Comput Biol. 2024 Jan 5;20(1):e1011759. doi: 10.1371/journal.pcbi.1011759 (PMC10795988; doi:10.1371/journal.pcbi.1011759)
Supplement: S5 Text — (DOCX) [file pcbi.1011759.s005.docx]

**Supplementary Materials for**

Detecting shifts in nonlinear dynamics using Empirical Dynamic Modeling with Nested-Library Analysis

Yong-Jin Huang, Chun-Wei Chang*, and Chih-hao Hsieh

*Correspondence to: [cwchang@ntu.edu.tw](mailto:cwchang@ntu.edu.tw)

**This supplement file includes:**

**S5 Text**

**S5 Text Application of NLA on different system variables of the food chain model**

# In Sec. 3.1, we apply our detection method on the variable $\boldsymbol{y}$ of the food-chain model. Here, we show the results when the same experiment is done using the system variable $\boldsymbol{x}$ and $\boldsymbol{z}$ instead. With Fig A, we can again conclude that NLA better detects and estimates the occurrence of regime shift when analyzing the system variable $\boldsymbol{x}$. When the input is replaced with the time series data of $\boldsymbol{z}$, CPMs based on Student-*t* test and Mann–Whitney test give good estimations. Comparatively, NLA is more accurate but less precise. The results of the experiments done with each of system variables are summarized in Table 1. The comparison with CPMs suggests that NLA is less dependent of the choice of input variable, and the performance is good in general.


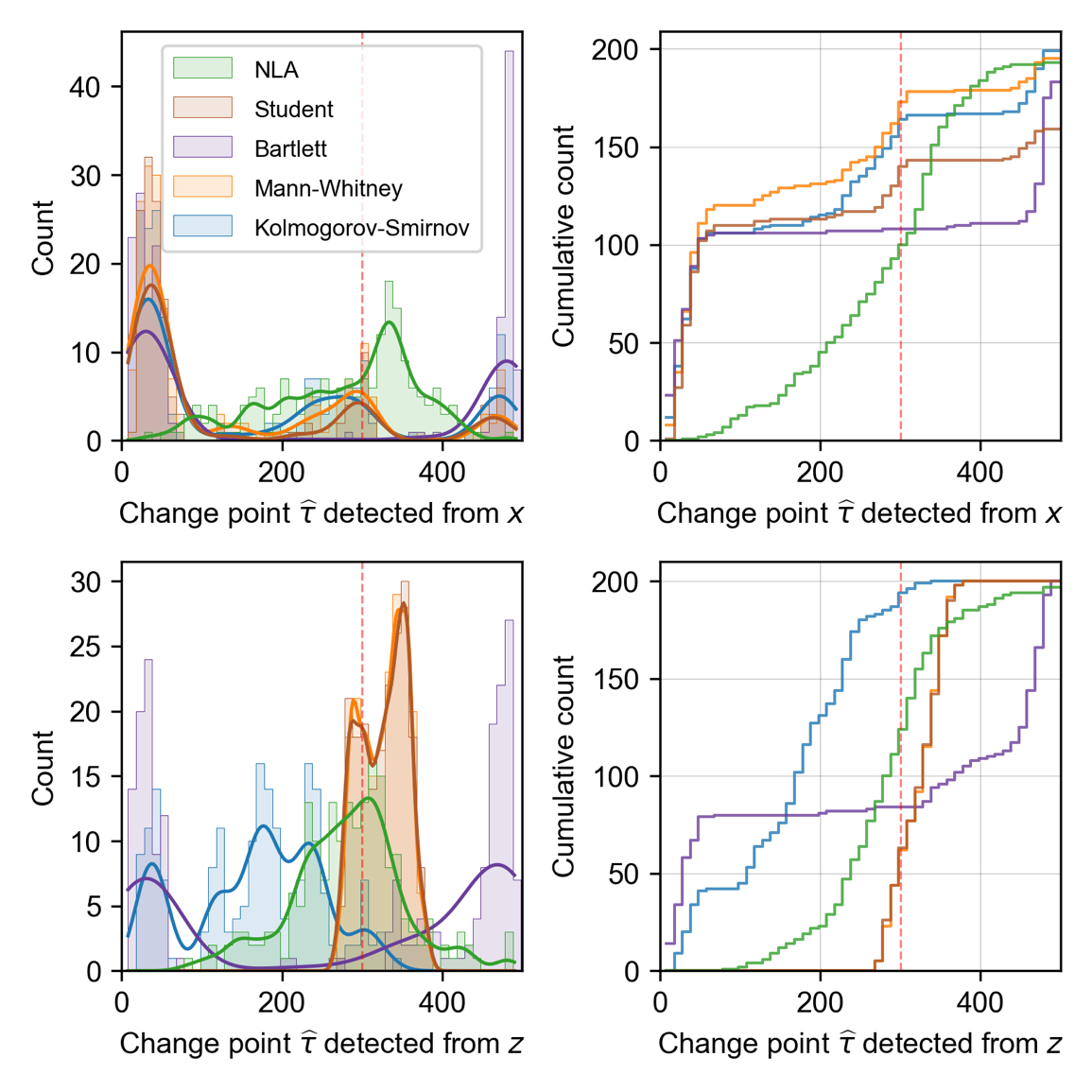


**Fig A:** The comparison between NLA and CPMs on *x* and *z*.
