# Supplementary material for: Detecting shifts in nonlinear dynamics using Empirical Dynamic Modeling with Nested-Library Analysis
Source: PLoS Comput Biol. 2024 Jan 5;20(1):e1011759. doi: 10.1371/journal.pcbi.1011759 (PMC10795988; doi:10.1371/journal.pcbi.1011759)
Supplement: S6 Text — (DOCX) [file pcbi.1011759.s006.docx]

**Supplementary Materials for**

Detecting shifts in nonlinear dynamics using Empirical Dynamic Modeling with Nested-Library Analysis

Yong-Jin Huang, Chun-Wei Chang*, and Chih-hao Hsieh

*Correspondence to: [cwchang@ntu.edu.tw](mailto:cwchang@ntu.edu.tw)

**This supplement file includes:**

**S6 Text**

**S6 Text Efficacy of NLA in analyzing time series underwent flickering regime shift**

We examined the efficacy of NLA method in analyzing the time series manifesting flickering regime shift in which there is no point-wise change point but a rather long transient period between two regimes (**Fig A**). We analyzed the model time series generated by the flickering model demonstrated in the previous work [1]. The simulation of flickering model repeated in 200 replicates. The upper panel of **Fig A** presents the computed median (blue line), 95%, and 5% quantiles (grey lines) of the time series replicates. Based on the computed median, the flickering dynamics occurred roughly between *t*=150 and *t*=350 and the red dashed line labeled the mid-point of the transient period at *t*=250. When applying NLA on the 200 time series replicates, the detected change points mostly located within the flickering period, indicating the effectiveness of NLA method in revealing the regime shift with long transient dynamics.


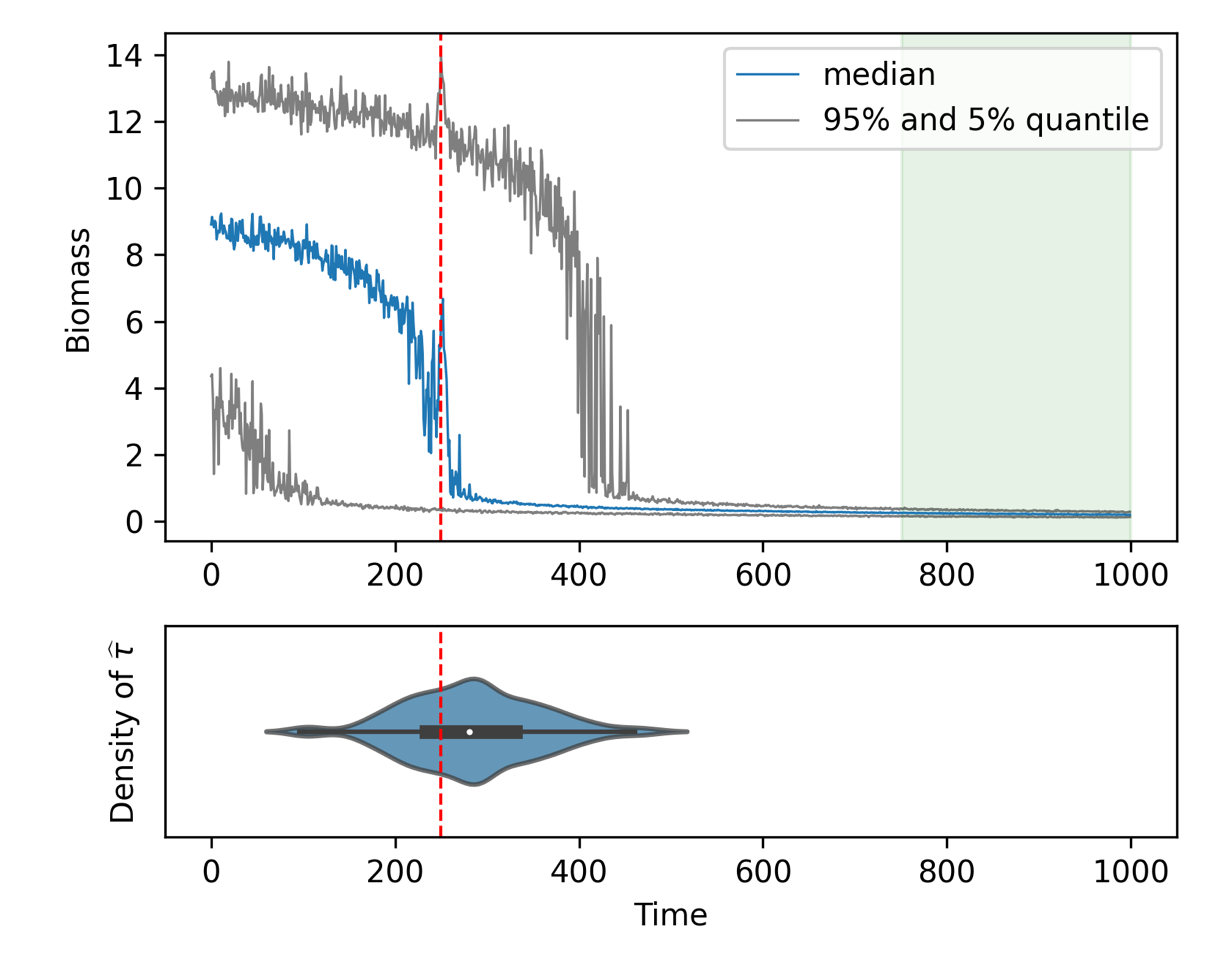


**Fig A**: NLA applied to the time series underwent flickering regime shift.

**References**

1. Dakos V, Carpenter SR, Brock WA, Ellison AM, Guttal V, Ives AR, et al. Methods for detecting early warnings of critical transitions in time series illustrated using simulated ecological data. PLoS ONE. 2012;7(7):e41010-e. Epub 2012/07/17. doi: 10.1371/journal.pone.0041010. PubMed PMID: 22815897.
