# Supplementary material for: Detecting shifts in nonlinear dynamics using Empirical Dynamic Modeling with Nested-Library Analysis
Source: PLoS Comput Biol. 2024 Jan 5;20(1):e1011759. doi: 10.1371/journal.pcbi.1011759 (PMC10795988; doi:10.1371/journal.pcbi.1011759)
Supplement: S7 Text — (DOCX) [file pcbi.1011759.s007.docx]

**Supplementary Materials for**

Detecting shifts in nonlinear dynamics using Empirical Dynamic Modeling with Nested-Library Analysis

Yong-Jin Huang, Chun-Wei Chang*, and Chih-hao Hsieh

*Correspondence to: [cwchang@ntu.edu.tw](mailto:cwchang@ntu.edu.tw)

**This supplement file includes:**

**S7 Text**

**S7 Text Efficacy of NLA in sparse time series**

We examined the efficacy of NLA method based on a model time series composed of more sparse data points. We reduced the sample size of model time series into a half of that used in original analysis (**Fig 3**) by doubling the sampling intervals. The result based on analyzing the sparse dataset (**Fig A**) indicates that change point can still be correctly detected from the valley of prediction error. However, compared to the findings using full sample size, the shape of prediction valley is shallower than that presented in **Fig 3**, suggesting the signal of change point became weaker when the time series becomes sparse.


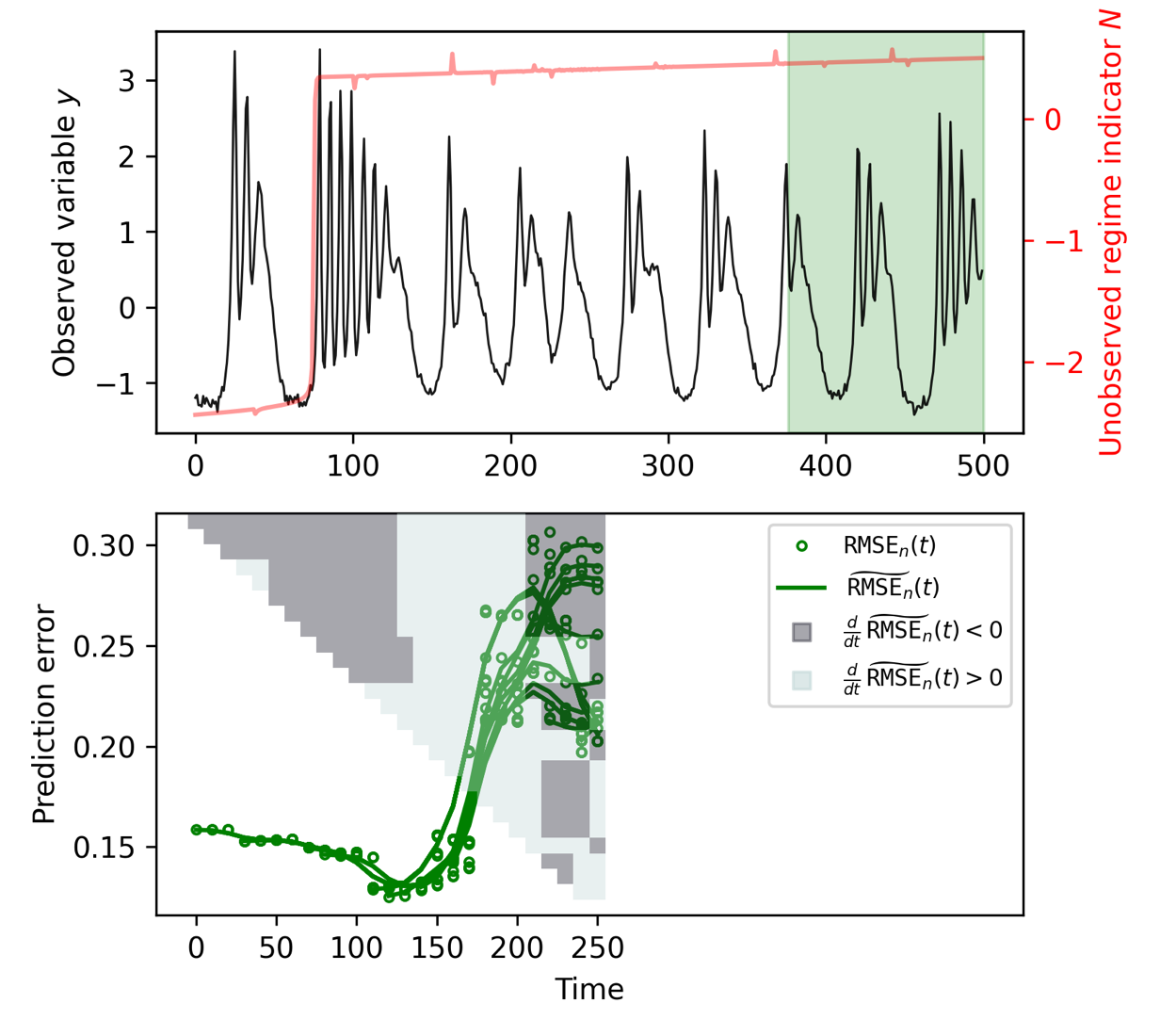


**Fig A**: NLA applied to the time series of *y* based on half of the sample size (an analogue of **Fig 3**).
