# Supplementary material for: Detecting shifts in nonlinear dynamics using Empirical Dynamic Modeling with Nested-Library Analysis
Source: PLoS Comput Biol. 2024 Jan 5;20(1):e1011759. doi: 10.1371/journal.pcbi.1011759 (PMC10795988; doi:10.1371/journal.pcbi.1011759)
Supplement: S8 Text — (DOCX) [file pcbi.1011759.s008.docx]

**Supplementary Materials for**

Detecting shifts in nonlinear dynamics using Empirical Dynamic Modeling with Nested-Library Analysis

Yong-Jin Huang, Chun-Wei Chang*, and Chih-hao Hsieh

*Correspondence to: [cwchang@ntu.edu.tw](mailto:cwchang@ntu.edu.tw)

**This supplement file includes:**

**S8 Text**

**S8 Text Robustness of NLA to the selection of test set located at either ends of the time series.**

NLA was applied to the model time series generated by the simulation stated in the Appendix C. To make a fair comparison, we included earlier time points that leads to a symmetric arrangement of the two test sets. We obtained the sampling distribution of the change point estimates by repeating the two NLA analyses on 150 replicates. Our results (**Fig A**) indicate that the sampling distributions obtained from forecasting the first quarter (blue) and last quarter (orange) are very close to each other. The sampling distribution considering the differences between the two estimates obtained from the same replicate also locates around zero, indicating robustness of NLA regardless of test set location.


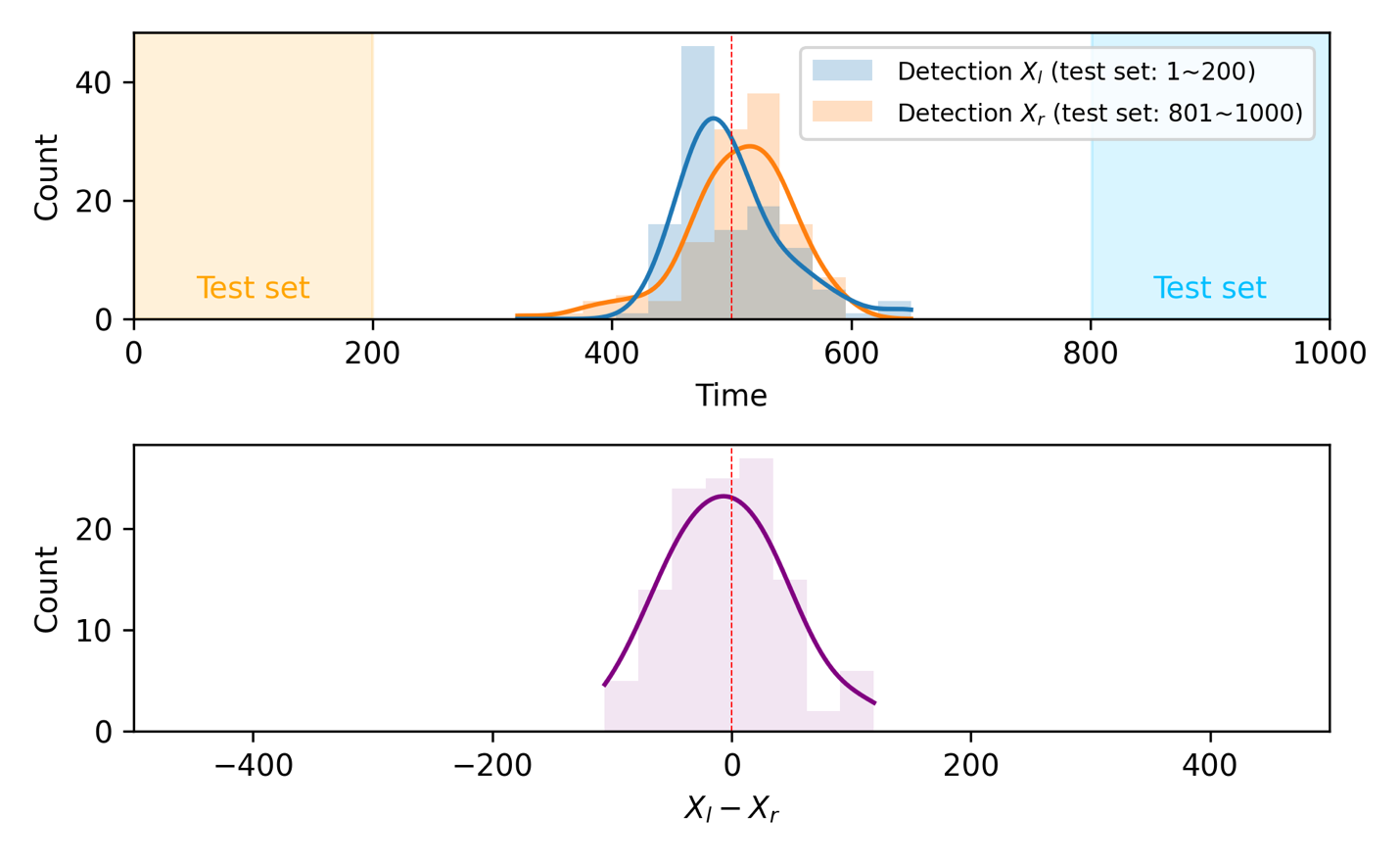


**Fig A**: Sampling distribution of change point estimates based on NLA analyses that allocate test sets in the first and last quarters.
